# Antidepressant Use During Acute Inpatient Care Is Associated With an Increased Risk of Psychiatric Rehospitalisation Over a 12-Month Follow-Up After Discharge

**DOI:** 10.3389/fpsyt.2019.00079

**Published:** 2019-02-22

**Authors:** Michael P. Hengartner, Silvia Passalacqua, Andreas Andreae, Thomas Heinsius, Urs Hepp, Wulf Rössler, Agnes von Wyl

**Affiliations:** ^1^Department of Applied Psychology, Zurich University of Applied Sciences, Zurich, Switzerland; ^2^Integrated Psychiatric Clinic of Winterthur and Zurich Unterland (ipw), Winterthur, Switzerland; ^3^Department of Psychiatry, Psychotherapy and Psychosomatics, University of Zurich, Zurich, Switzerland; ^4^Laboratory of Neuroscience (LIM 27), Institute of Psychiatry, University of São Paulo, São Paulo, Brazil; ^5^Department of Psychiatry and Psychotherapy, Charité—Universitätsmedizin Berlin, Berlin, Germany

**Keywords:** antidepressants, affective disorders, depression, outcome, rehospitalisation, propensity score

## Abstract

**Background:** Some evidence suggests that antidepressants may relate to poor outcomes in depression. The aim of this study was, therefore, to examine, whether antidepressant use may worsen the long-term outcome in real-world psychiatric patients with both primarily affective and non-affective mental disorders.

**Methods:** Based on a total of *n* = 151 inpatients with a mixed range of diagnoses enrolled at two psychiatric hospitals in Zurich, Switzerland, matched pairs of *n* = 45 antidepressant users and *n* = 45 non-users were selected via nearest neighbor propensity score matching. Pairs were matched according to 14 clinically relevant covariates assessing psychosocial impairments, functioning deficits and illness severity. The two outcomes of interest were the number and total duration of all rehospitalisations over a 12-month follow-up after discharge from the hospital based on the official clinical registry.

**Results:** Altogether 35.6% of antidepressant users were rehospitalised at least once, as compared to 22.2% in matched non-users. Two or more rehospitalisations occurred in 22.2% of antidepressant users but only in 2.2% of non-users. In antidepressant users, the mean total duration of rehospitalisations was 22.22 days, as compared to 8.51 in matched non-users. According to Poisson regression analyses, antidepressant use during acute inpatient care prospectively relates to both a higher risk (incidence rate ratio [IRR] = 3.64, 95% confidence interval [95%-CI] = 1.71–7.75, *p* = 0.001) and a longer duration (IRR = 2.61, 95%-CI = 1.01–6.79, *p* = 0.049) of subsequent rehospitalisations. These findings were consistently replicated when traditional multivariable regression analysis was applied to the full sample. Findings also replicated when patients with affective and non-affective disorders were analyzed separately.

**Conclusions:** Our findings raise the possibility that, in the long-term, antidepressants may impair recovery and increase the risk of rehospitalisation in patients with both primarily affective and non-affective disorders. More work is required to explore possible aetiopathological pathways leading to psychiatric rehospitalisation.

## Introduction

The persistent increase in antidepressant drug prescriptions over the last three decades left the global prevalence of major depression and anxiety disorders largely unchanged (1), whereas disability owing to affective disorders has even increased on a population level (2, 3). This is a paradox if we assume that antidepressants effectively facilitate remission and prevent relapse in the long-term. As a result, the long-term benefits of antidepressant pharmacotherapy have been contested (4–6). In the following we will provide are brief overview of the pertinent literature on this controversial issue.

### Evidence From Discontinuation Trials

The evidence for long-term benefits of antidepressants relies mainly on discontinuation trials, in which remitted/recovered patients on antidepressants are randomized to either abrupt switching to placebo (i.e., treatment discontinuation) or continuing drug use (i.e., maintenance therapy). These discontinuation trials indeed suggest that antidepressants may prevent depression relapse [for a recent meta-analysis, see for instance (7)], but the validity of these studies is limited. First, they include only patients who responded to acute drug treatment, hence, the results do not apply to the many patients who recover spontaneously or to patients who do not respond to the drugs. Second, the control group consists of drug-responders who were randomized to have the drug discontinued rapidly and replaced by placebo, hence placebo-controls may experience withdrawal syndromes which may be misdiagnosed as depression relapse or which themselves may cause relapse due to their distressing nature (5, 6, 8). Several authors have therefore suggested that higher relapse rates after antidepressant discontinuation, relative to continuing pharmacotherapy, are due to oppositional tolerance, which describes an iatrogenic process of neurobiological adaptation to prolonged pharmacological perturbation (9–11). Consistent with the hypothesis that relapse rates in discontinuation trials are mostly due to withdrawal reactions, it has been shown that the relative risk of relapse is highest during the first 1–3 months after drug discontinuation; afterwards there is no risk difference between those patients who stayed on the drugs and those who were switched to placebo (8, 12, 13). Therefore, and although recommendation for maintenance pharmacotherapy is largely based on discontinuation trials, they cannot inform whether antidepressant users, as compared to non-users, have a better long-term outcome.

### Evidence From Other Long-Term Trials and From Observational Studies

In the following we will therefore briefly summarize the evidence from other research designs, including placebo-controlled parallel-arm long-term trials and prospective observational studies. In two large pragmatic real-world trials based on representative samples of patients with major depression, only about 5% of continuously drug-treated participants achieved sustained remission over 12 months (14, 15). That is, enduring symptom remission is rare whereas relapse is very common even in intensively drug-treated patients. Unfortunately, in these two trials referenced above there was no un-medicated control group, but mounting evidence suggests that untreated patients may not have a worse outcome. For instance, a meta-analysis of placebo-controlled long-term parallel-arm trials of 6–8 months duration for major depression revealed that antidepressant use may not improve remission rates (16). In another meta-analysis it was further shown that patients whose depression remitted while on placebo have a lower relapse risk upon treatment discontinuation than patients who were on antidepressants (9). What about evidence for conditions other than major depression? In a recent 20-week randomized trial of people with complicated grief, the outcome for antidepressants did not differ from placebo, but was significantly worse than the outcome for psychotherapy in terms of functional impairment, depression symptoms, and suicidality (17). Likewise, some long-term follow-ups of clinical trials for anxiety disorders suggest that, relative to placebo (18) or psychotherapy (19), antidepressants may impair recovery and worsen the long-term outcome.

Of course this brief overview was not exhaustive, but it demonstrates that there is compelling evidence from many randomized controlled trials that antidepressants may not improve long-term outcomes (5, 6). Unfortunately, all these clinical trials (and their naturalistic follow-ups) either relied on pre-selected, unrepresentative samples [e.g., (16, 19)] or did not include an un-medicated control-group [e.g., (14, 15)]. Therefore, naturalistic observational studies based on real-world patient samples drawn from routine care services and from the general population are required. Such studies commonly suggest that in people with affective disorders, antidepressant use, compared to non-use, relates to a poorer long-term outcome (20–26). These findings are frequently met with disbelief by many experts in psychiatry. A common objection to findings from observational studies therefore is that they are unreliable due to selection bias. However, this assumption is empirically unfounded. Meta-analytic research that systematically compared treatment outcomes from observational studies with those from randomized trials does not indicate that treatment outcomes differ by research design (27, 28).

### The Present Study

Nevertheless, naturalistic observational studies certainly have limitations. So far, most studies focused exclusively on patients with major depression, and there is a lack of research on the long-term outcome of antidepressant use in patients with primarily non-affective mental disorders. Moreover, in previous research on the outcome of antidepressant pharmacotherapy the main outcome was typically based on assessments with subjective depression rating-scales, which may have limited validity (29, 30). Symptom rating scales may also fail to adequately capture clinically significant functional outcomes such as rehospitalisation and long-term disability. Confounding by indication is another problem that threatens the validity of observational studies (31). Although recent work has tried to address this problem by including potential confounders as covariates in multivariable regression models, such an approach may not be sufficient. Selection bias is more adequately accounted for with propensity score methods (32). In the present study we will therefore focus on rehospitalisation rates derived from a clinical registry, which is an objective and unbiased outcome. Moreover, we will include patients with both primarily affective and non-affective mental disorders and use propensity score matching analysis to minimize selection bias.

The aim of the present work was thus to explore, whether antidepressant use during an acute psychiatric inpatient hospitalization predicts rehospitalisation rates over a 12-month follow-up after discharge. Based on our previous analysis of a prospective community sample (26), we hypothesized that antidepressant use would prospectively relate to higher rehospitalisation rates.

## Methods

### Design and Procedure

We used data from the Post-Discharge Network Coordination Programme (PDNC-P). The methods of this randomized controlled trial have been described in detail elsewhere (33, 34). In short, the trial was designed to test the outcome of a brief case management intervention carried out by social workers. The aim of this trial was to reduce rehospitalisation rates and to increase long-term mental health and functioning in psychiatric inpatients without a history of repeated hospitalisations. The inclusion criteria were: (1) no more than three hospitalisations within the last 3 years (including the index hospitalization), (2) a Global Assessment of Functioning (GAF) score of 60 or lower, (3) cognitive ability to provide written informed consent, and (4) age between 18 and 64 years. Exclusion criteria were: (1) insufficient German language proficiency, (2) simultaneous support by another case manager, and (3) living in supportive housing. The PDNC-P comprised two treatment arms: Half of the participants were randomly assigned to a brief case management intervention detailed in Hengartner et al. (35) and the other half received treatment as usual (i.e., no coordinated support after discharge). Both groups were assessed during acute inpatient care (t_0_), 3 months after discharge when the intervention terminated (t_1_), and 12 months after discharge (t_2_). Participants and assessors were blind toward group allocation at baseline measurement t_0_. The recruitment began in September 2011 and the last follow-up assessment of t_2_ was carried out in April 2015. Rehospitalisations at 12-month follow-up (t_2_) were assessed for the full sample based on the clinical registry.

The trial was approved by the cantonal ethics committee of Zurich (reference number: KEK-ZH 2011-0175) and pre-registered in the International Standard Randomized Controlled Trial Number (ISRCTN) register (reference number: ISRCTN58280620). The study protocol was published freely available online (34).

### Participants

The PDNC-P included 151 inpatients from the Winterhur—Zurich Unterland psychiatric catchment area, an urban/suburban area of high-level resources near the city of Zurich, Switzerland. For more information, see references (33, 34). The participants were enrolled at two different psychiatric hospitals: the Psychiatrie-Zentrum Hard in Embrach and the Klinik Schlosstal in Winterthur, which are both part of the umbrella organization Integrierte Psychiatrie Winterthur—Zürcher Unterland (IPW). The sample comprised 79 men (51.7%) and 72 women (48.3%). Their mean age was 41.6 years (*SD* = 11.3) and ranged from 18 to 61 years. For *n* = 85 (56.3%) it was the first hospitalization, for *n* = 45 (29.8%) the second and for *n* = 21 (13.9%) the third hospitalization. A total of *n* = 37 (24.5%) had a primary diagnosis of substance-use disorder (SUD; ICD-10 code F10-F19), *n* = 41 (27.2%) of schizophrenia and other psychotic disorders (F20-F29), *n* = 52 (34.4%) of a mood disorder (F30-F39, whereof *n* = 34 had a depressive disorder, F32 or F33), and *n* = 21 (13.9%) had other disorders (whereof *n* = 17 had an anxiety and stress-related disorder F41-F43; *n* = 3 a personality disorder F60; and *n* = 1 an attention deficit hyperactivity disorder F90). The three patients with personality disorders and the one patient with attention deficit hyperactivity disorder all had comorbid affective disorders (F32 and/or F43). As a result, *n* = 78 patients (51.7%) were broadly classified with a primarily non-affective disorder (comprising SUD and psychotic disorders) and *n* = 73 (48.3%) with an affective disorder (comprising mood, anxiety and stress-related disorders). A total of *n* = 39 (25.8%) were prescribed a SSRI, *n* = 11 (7.3%) with a TCA, and *n* = 11 (7.3%) other antidepressants. During the index hospitalization altogether *n* = 54 (35.8%) used an antidepressant, *n* = 48 (31.8%) used neuroleptics, and *n* = 16 (10.6%) concurrently used both antidepressants and neuroleptics. All antidepressant users were discharged from the hospital with a continued antidepressant prescription.

### Outcomes and Measures

Primary outcomes in the PDNC-P as well as in the present study were the frequency and the duration of rehospitalisations over the 12-month observation period following discharge as assessed with the IPW clinical registry (t_2_ assessment). Frequency of rehospitalisations was defined as the total number of readmissions, whereas duration of rehospitalisations was defined as the sum of all inpatient days over all readmissions. For instance, when a patient was rehospitalised twice, the first time for 10 days and the second time for 20 days, then his/her number of rehospitalisations was 2 and the total duration of rehospitalisations was 30 days. Another patient may also have 2 rehospitalisations, the first for 20 days and the second for 30 days, which adds up to a total of 50 days. That is, although both exemplary patients had 2 rehospitalisations, they differed in their total length of rehospitalisations. Since all psychiatric hospitalisations within the IPW catchment area are recoded in the clinical registry, there were no missing data.

Antidepressant use and socio-demographics were assessed with the Client Socio-Demographic and Service Receipt Inventory—European Version [CSSRI-EU; (36)] during acute inpatient care (t_0_ assessment). We further included the following variables assessed during acute inpatient care (t_0_ assessment): A patients' functioning at baseline was rated by a blinded assessor with the Social and Occupational Assessment Scale [SOFAS; (37)] as well as with the GAF score (38). Social support was measured with the “Fragebogen zur sozialen Unterstützung—Kurzform 14” [F-SozU; (39)]. The F-SozU is a German self-rating questionnaire comprising items from the following three domains of perceived social support: emotional support, instrumental support, and social integration. Psychopathology and illness severity were assessed via assessor-rating using the Health of the Nation Outcome Scales [HoNOS; (40)] as well as via patients' self-rating using the Outcome Questionnaire 45 [OQ-45, German version; (41)]. Finally, subjective quality of life was rated by the patients with the Manchester Short Assessment of Quality of Life [MANSA; (42)].

### Statistical Analysis

We extracted matched pairs of antidepressant users and non-users based on propensity score analysis (43). As recommended in the literature (32), we used nearest neighbor matching based on logistic regression and a maximal caliper distance of 0.2. Propensity score matching assigns a control subject to an experimental subject via the multivariate analysis of covariates. By this means antidepressant users are compared to non-users that are similar with respect to various clinically relevant variables that were selected a priori. We included the following 14 covariates: sex (men vs. women), age (in years), relationship status (single vs. in relationship) education level (high vs. other), receipt of state benefits (yes vs. no), first hospitalization at index (yes vs. no), primary disorder (affective vs. non-affective), neuroleptic use (yes vs. no), assessor-rated severity of psychopathology (HoNOS), subjective severity of psychopathology (OQ45), perceived social support (FsozU), social functioning (SOFAS), global level of functioning (GAF), and subjective quality of life (MANSA). The case management intervention to which half of all participants were assigned in this trial had no effect on rehospitalisation rates and therefore was omitted from the analysis [for a detailed account, see reference (33)]. The prospective associations between antidepressant use during acute inpatient care (t_0_ assessment) and the frequency and duration of rehospitalisations within 12 months after discharge (t_2_ assessment) were examined with a series of Poisson regression analyses with log link-function. Poisson regression was chosen because both outcomes were right-skewed counts (i.e., non-negative integer values). Antidepressant use was entered as the predictor variable and both frequency and duration of rehospitalisations separately as the outcome variable. Findings of the Poisson regression analyses were reported with the incidence rate ratio (IRR), which is a convenient effect size to evaluate to effect of different predictor variables on Poisson-distributed outcomes. All analyses were conducted with SPSS version 24 for Windows.

## Results

Out of a base of *n* = 151 inpatients, comprising *n* = 54 (35.8%) antidepressant users and *n* = 97 (64.2%) non-users, propensity score matching analysis extracted 45 matched pairs (i.e., 45 antidepressant users and 45 non-users). The paired dataset included *n* = 45 men and women each with a mean age (SD) of 43.1 (10.8) years. A total of *n* = 49 (54.4%) inpatients had primarily an affective disorder (depression, anxiety, and stress-related disorders), and *n* = 41 (45.6%) a non-affective disorder (SUD and psychotic disorders). For *n* = 47 (52.2%) it was the first hospitalization in the last 3 years. Table 1 shows the distribution of the 14 covariates on which basis antidepressant users were matched to corresponding non-users. There were no differences between pairs of users and non-users and the two groups were perfectly balanced (overall balance test: χ^2^ = 1.626, df = 14, *p* = 1.000; number of unbalanced covariates: 0). A detailed description of specific primary diagnoses is provided in Table 2. A primary diagnosis of alcohol use disorder was more common among antidepressant users, whereas schizophrenia was more frequent among non-users, but both comparisons did not reach statistical significance after correcting for multiple testing (α = 0.005).

**Table 1:** Clinical and socio-demographic characteristics of antidepressant (AD) users and non-users.

		**No AD use (*n* = 45)**	**AD use (*n* = 45)**	***P***
Sex	Men	22 (48.9)	23 (51.1)	1.000 ^a^
	Women	23 (51.1)	22 (48.9)	
Age	Years	43.2 (11.4)	43.0 (10.4)	0.931 ^b^
Relationship status	Single	22 (51.2)	21 (48.8)	1.000 ^a^
	In relationship	23 (48.9)	24 (51.1)	
Education level	High	10(47.6)	11 (52.4)	1.000 ^a^
	Other	35 (50.7)	34 (49.3)	
Receipt of state benefits	Yes	25 (49.0)	26 (51.0)	1.000 ^a^
	No	20 (51.3)	19 (48.7)	
Index admission	First	24 (51.1)	23 (48.9)	1.000 ^a^
	Second or third	21 (48.8)	22 (51.2)	
Primary disorder	Affective	24 (49.0)	25 (51.0)	1.000 ^a^
	Non-affective	21 (51.2)	20 (48.8)	
Neuroleptic use	Yes	14 (50.0)	14 (50.0)	1.000 ^a^
	No	31 (50.0)	31 (50.0)	
Psychopathology (assessor-rated)	HoNOS	1.4 (0.5)	1.4 (0.4)	0.742 ^b^
Psychopathology (self-rated)	OQ45	78.3 (29.7)	77.7 (18.5)	0.909 ^b^
Perceived social support	FsozU	3.6 (0.9)	3.6 (0.9)	0.843
Social functioning	SOFAS	42.8 (13.1)	41.4 (11.1)	0.592
Global functioning	GAF	36.0 (12.5)	34.8 (10.3)	0.639
Subjective quality of life	MANSA	4.3 (1.2)	4.2 (0.9)	0.697

*Categorical variables are reported with number and percent (in brackets) and continuous variables with mean and standard deviation (in brackets)*.

a *Fisher's Exact Test (two-sided)*;

b *Independent-samples T-Test (two-sided)*.

**Table 2:** Number and frequency (in brackets) of primary diagnoses in antidepressant (AD) users and non-users in the propensity score matched sample (*n* = 90).

	**No AD use (*n* = 45)**	**AD use (*n* = 45)**	***P***
Alcohol use disorder (F10)	4 (8.9)	12 (26.7)	0.027
Polysubstance abuse (F19)	1 (2.2)	1 (2.2)	1.0
Schizophrenia (F20)	11 (24.4)	3 (6.7)	0.020
Acute psychotic disorder (F23)	2 (4.4)	1 (2.2)	0.557
Schizoaffective disorder (F25)	2 (4.4)	2 (4.4)	1.0
Bipolar disorder (F31)	8 (17.8)	4 (8.9)	0.215
Depression episode (F32)	4 (8.9)	8 (17.8)	0.215
Recurrent depression (F33)	5 (11.1)	4 (8.9)	0.725
Panic disorder (F41)	1 (2.2)	2 (4.4)	0.557
Severe stress and adjustment disorder (F43)	4 (8.9)	4 (8.9)	1.0

**Figure 1:**
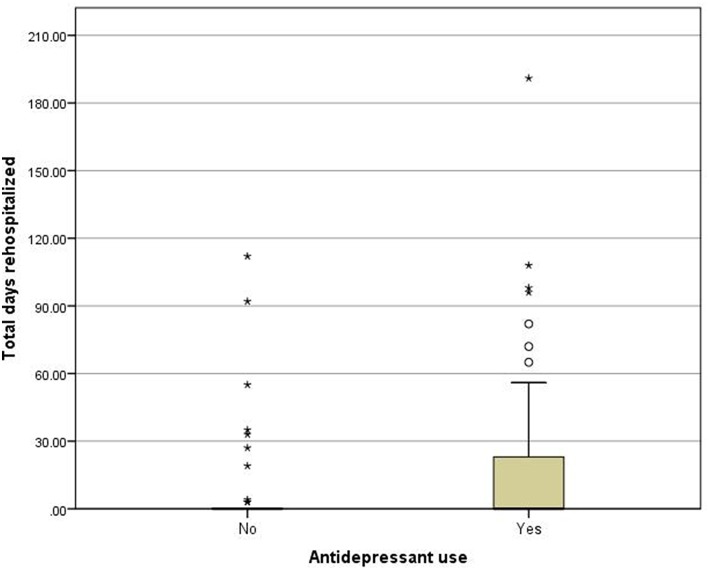
Boxplots for total duration of rehospitalizations (in days) for antidepressant users and non-users. Asterisk indicate single outliers.

In antidepressant users (*n* = 45), the number of rehospitalisations ranged from 0 to 6. A total of 16 patients (35.6%) had at least 1 rehospitalisation, 10 (22.2%) had 2 or more rehospitalisations and 7 patients (15.6%) had 3 or more rehospitalisations. In the matched non-users (*n* = 45), the number of rehospitalisations ranged from 0 to 2 and 10 patients (22.2%) had at least 1 rehospitalisation. Only 1 patient (2.2%) had 2 rehospitalisations. A detailed account is provided in Table 3. The total duration of rehospitalisations ranged from 0 to 191 days, with a mean (SD) 22.22 (40.61) in antidepressant users, and from 0 to 112, with a mean (SD) of 8.51 (23.46) in non-users. The corresponding boxplots are shown in Figure 1. The results of the Poisson regression analysis showed that antidepressant use was prospectively associated with a significantly increased number (IRR = 3.64, 95%-CI = 1.71-7.75, *p* = 0.001) and duration (IRR = 2.61, 95%-CI = 1.01-6.79, *p* = 0.049) of subsequent rehospitalisations.

**Table 3:** Number of psychiatric rehospitalisations within 12-months after discharge from the index hospitalization in *n* = 45 antidepressant users and *n* = 45 propensity-score matched non-users.

	**Number of rehospitalisations**	**Frequency**	**Percent**	**Cumulative Percent**
AD users	0	29	64.4	64.4
	1	6	13.3	77.8
	2	3	6.7	84.4
	3	3	6.7	91.1
	4	2	4.4	95.6
	5	1	2.2	97.8
	6	1	2.2	100
Non-users	0	35	77.8	77.8
	1	9	20.0	97.8
	2	1	2.2	100

As part of a sensitivity analysis we aimed at reproducing our results with traditional multivariable regression analysis in the full sample. For it we conducted two Poisson regression analyses, first with number of rehospitalisations as the outcome and second with duration of rehospitalisations as the outcome. In both models we included antidepressant use as the main predictor variable and the following potential confounders as control variables: sex, age, education level, relationship status, receipt of state benefits, primary diagnosis, neuroleptic use, number of index admission, other-rated severity of psychopathology (HoNOS), global level of functioning (GAF), subjective quality of life (MANSA), social functioning (SOFAS), self-rated severity of psychopathology (OQ45 sum score), and perceived social support (FsozU). Due to some missing values in the covariates, *n* = 131 patients, whereof *n* = 49 antidepressant users, were included in this analysis. Consistent with our propensity score matching analysis, antidepressant use related to both a higher number of rehospitalisations (IRR = 3.71, 95%-CI = 1.87-7.36, *p* < 0.001) and to a longer duration of rehospitalisations (IRR = 6.14, 95%-CI = 2.25-16.74, *p* < 0.001). When patients with bipolar disorder were excluded, *n* = 44 antidepressant users and *n* = 73 non-users remained in the dataset. A re-analysis with this restricted sample (*n* = 117) yielded similar results (for number of rehospitalisations: IRR = 4.28, 95%-CI = 2.10-8.70, *p* < 0.001; for duration of rehospitalisations: IRR = 6.16, 95%-CI = 2.42-15.72, *p* < 0.001).

We also re-conducted our analysis stratified according to primary diagnostic group. In patients with non-affective primary diagnosis (*n* = 67, whereof *n* = 21 antidepressant users), controlling for all other covariates, antidepressant use related to both higher number (IRR = 3.70, 95%-CI = 1.87–7.34, *p* < 0.001) and longer duration of rehospitalisations (IRR = 6.10, 95%-CI = 1.67–22.22, *p* = 0.006). Likewise, in patients with an affective primary diagnosis (*n* = 64, whereof *n* = 28 antidepressant users), antidepressants related to both higher number (IRR = 3.47, 95%-CI = 1.34–9.00, *p* = 0.010) and longer duration of rehospitalisations (IRR = 8.25, 95%-CI = 2.78–24.47, I, *p* < 0.001).

## Discussion

### Summary

In this prospective naturalistic observational study we related antidepressant use during acute hospital care to subsequent rehospitalisations over a 12-month follow-up after discharge in a propensity score matched sample of *n* = 90 psychiatric inpatients with primarily affective (mood, anxiety, and stress-related disorders) and non-affective disorders (substance-use and psychotic disorders). The results indicate that antidepressant use during acute inpatient care relates to 3.5-fold increased risk of experiencing a rehospitalisation and to a 2.5-fold increased duration of total days rehospitalised over 12 months. The results were replicated when instead of propensity score matching analysis a less stringent, traditional multivariable regression analysis was applied to the full sample of *n* = 131 participants. Moreover, all effects were replicated when patients with bipolar disorder were excluded from the analysis and when affective and non-affective disorders were analyzed separately.

### Strengths and Limitations

The strengths of the present work comprise its prospective design, thorough assessment of various clinically important covariates and an unbiased, objective study outcome. The main improvement over previous observational studies is the sophisticated statistical adjustment for selection bias (i.e., confounding by indication). Based on a nearest neighbor propensity score matching analysis we selected a group of antidepressant users that was perfectly matched to non-users based on 14 clinically important covariates. By this means we were able to minimize confounding by indication in non-random treatment groups (32). Although many researchers and clinicians falsely believe that the results of observational studies are not reliable, it is important to emphasize that healthcare outcomes assessed with observational studies do not differ systematically from those assessed in randomized clinical trials (27, 28).

Nevertheless, our study has important limitations, which we critically acknowledge before we proceed with a thorough discussion of our findings. First, and most importantly, patients were not randomly assigned to antidepressant use, which precludes causal conclusions. Although we matched drug-users with similar non-users based on 14 covariates, including psychosocial impairments, illness severity and functioning deficits, we cannot rule out that there are unmeasured confounders which introduced selection bias. Second, with *n* = 90 the propensity score matched sample was rather modest. Future studies with larger samples are necessary to increase power and precision. Third, we did not assess for how long the patients continued antidepressant use after discharge from the hospital. Fourth, we do not know for which reasons participants were rehospitalised. The specific cause of rehospitalisation could help to work out possible etiological mechanisms linking antidepressant use to the re-occurrence of acutely distressing psychopathology.

### Interpretation of Findings

Several long-term trials suggest that antidepressants may not produce clinically meaningful long-term benefits (14–16). In accordance with mounting evidence from observational studies, our findings even raise the possibility that antidepressant pharmacotherapy may increase relapse rates and impair recovery in the long run (20, 21, 23, 24, 26). These findings are in accord with a comprehensive meta-analysis of long-term clinical trials by Andrews et al. (9), which shows that patients with major depression who recovered on placebo have a lower relapse rate upon treatment discontinuation than patients who recovered on drugs. These findings conflict with the results from discontinuation trials (7), which aim at estimating long-term relapse prevention in continuously drug-treated patients compared to patients whose medication was discontinued rapidly and replaced by placebo.

Although recommendation for maintenance therapy is mostly based on these discontinuation trials, their validity is poor due to several limitations (5, 6). Since antidepressant discontinuation can cause severe withdrawal reactions, including depression-like symptoms such as lethargy, fatigue, irritability, suicidal ideation, and sleep problems (44), withdrawal reactions are easily misdiagnosed as depression relapse (8, 13, 44). The higher relapse rates in participants switched to placebo could thus be the result of neurobiological adaptations due to prior pharmacotherapy, a condition that was referred to as oppositional tolerance (4, 9). Moreover, preventive effects reported in discontinuation trials apply only to patients who favorably respond to antidepressant, but not to non-responders and spontaneous remitters (16).

As there appears to be an association between antidepressant use and rehospitalisation rates, it is important to propose plausible causal mechanisms that may account for such a relationship. Although we were not able to assess these mechanisms in our study, possible pathways have been described in the literature. Owing to pharmacodynamic alterations in neurobiological functions, including reduction of receptor density in response to increased neurotransmitter concentration (45), affective and psychosomatic disturbances following long-term antidepressant use are possibly iatrogenic, that is, attributable to drug-induced neurobiological adaptations (4, 9, 10). These treatment-emergent adverse effects of psychotropic medications may develop following antidepressants discontinuation of mostly long-term use and in some patients withdrawal is severe to an extent that they develop protracted post-withdrawal affective disorders (44, 46). In other instances continuing antidepressant therapy after initial response may cause new mental disorders, which has also been referred to as behavioral toxicity (46).

Meta-analyses of placebo-controlled randomized trials indeed suggest that some rehospitalisations in treatment-adherent antidepressant users are possibly due to adverse drug reactions. For instance, in patients with mood and anxiety disorders, antidepressants may trigger psychomotor agitation and mania (47, 48) or suicidal acts (49, 50). Moreover, in healthy volunteers without mental disorders, relative to placebo, antidepressant use has been associated with a significantly increased risk of adverse mental events, including agitation, sleep problems, nervousness, and abnormal thinking (51, 52). These iatrogenic psychological disturbances may possibly account for the increased rate and duration of rehospitalisations observed in the present study, but more work is required to establish causal aetiopathological mechanisms.

## Conclusions

Our data suggest that antidepressant use during acute inpatient care, compared to non-use, may increase the risk and duration of subsequent rehospitalisations over a 12-month follow-up in patients with both primarily affective and non-affective disorders. Our findings therefore challenge the alleged long-term benefit of antidepressants and raise the possibility, that, in the long run, antidepressants may possibly do more harm than good (4, 5, 53, 54). The naturalistic design of our study does not allow for causal conclusions, yet we suggest that it provides a signal that warrants more research. In particular, we suggest that more attention should be paid to possible iatrogenic effects of long-term antidepressant pharmacotherapy, which still is an insufficiently researched topic. Future work should explore potential causal pathways, such as for instance agitation, emotional numbing and/or neurobiological susceptibility to acute stress, through which antidepressant use may impair long-term functioning and well-being. Severe and sometimes protracted withdrawal reactions after drug taper are another important but under-researched topic. That is, higher rehospitalisation rates in antidepressant users could be due to adverse reactions to long-term antidepressant use (10) or the result of severe withdrawal reactions upon drug discontinuation (44). These potential mechanisms are tentative and still inconclusive, but they provide an important avenue for future research into the long-term effects of antidepressants.

## Informed consent

Informed consent was obtained from all individual participants included in the study.

## Author Contributions

MH drafted the manuscript and conducted all statistical analyses. SP contributed to data collection and critical revision of the manuscript. AA, WR, and AvW contributed to design and conduct of the study, interpretation of the data, and critical revision of the manuscript. TH and UH contributed to interpretation of the data and critical revision. All authors critically revised the manuscript and approved the final version of this manuscript.

### Conflict of Interest Statement

The authors declare that the research was conducted in the absence of any commercial or financial relationships that could be construed as a potential conflict of interest.
